# Glutathione S-Transferase (GST) Gene Diversity in the Crustacean *Calanus finmarchicus* – Contributors to Cellular Detoxification

**DOI:** 10.1371/journal.pone.0123322

**Published:** 2015-05-06

**Authors:** Vittoria Roncalli, Matthew C. Cieslak, Yale Passamaneck, Andrew E. Christie, Petra H. Lenz

**Affiliations:** 1 Békésy Laboratory of Neurobiology, Pacific Biosciences Research Center, University of Hawaii at Manoa, Honolulu, Hawaii, United States of America; 2 Kewalo Marine Laboratory, Pacific Biosciences Research Center, University of Hawaii at Manoa, Honolulu, Hawaii, United States of America; University of South Florida College of Medicine, UNITED STATES

## Abstract

Detoxification is a fundamental cellular stress defense mechanism, which allows an organism to survive or even thrive in the presence of environmental toxins and/or pollutants. The glutathione S-transferase (GST) superfamily is a set of enzymes involved in the detoxification process. This highly diverse protein superfamily is characterized by multiple gene duplications, with over 40 GST genes reported in some insects. However, less is known about the GST superfamily in marine organisms, including crustaceans. The availability of two *de novo* transcriptomes for the copepod, *Calanus finmarchicus*, provided an opportunity for an in depth study of the GST superfamily in a marine crustacean. The transcriptomes were searched for putative GST-encoding transcripts using known GST proteins from three arthropods as queries. The identified transcripts were then translated into proteins, analyzed for structural domains, and annotated using reciprocal BLAST analysis. Mining the two transcriptomes yielded a total of 41 predicted GST proteins belonging to the cytosolic, mitochondrial or microsomal classes. Phylogenetic analysis of the cytosolic GSTs validated their annotation into six different subclasses. The predicted proteins are likely to represent the products of distinct genes, suggesting that the diversity of GSTs in *C*. *finmarchicus* exceeds or rivals that described for insects. Analysis of relative gene expression in different developmental stages indicated low levels of GST expression in embryos, and relatively high expression in late copepodites and adult females for several cytosolic GSTs. A diverse diet and complex life history are factors that might be driving the multiplicity of GSTs in *C*. *finmarchicus*, as this copepod is commonly exposed to a variety of natural toxins. Hence, diversity in detoxification pathway proteins may well be key to their survival.

## Introduction

The activation of multiple cellular stress defense mechanisms, including an increase in the activity of detoxification enzymes, is key to an organism’s ability to survive, and sometimes even thrive, in environments characterized by the presence of toxins and/or pollutants [1]. In eukaryotes, the cellular detoxification process can be divided into three phases [2]. In Phase I, reactive/polar groups are enzymatically added to a xenobiotic. In the second phase (Phase II), the modified toxicant is enzymatically conjugated to a polar molecule. In the final phase of the detoxification process (Phase III), efflux transporters that specifically recognize conjugated toxins remove the modified xenobiotic from the cell.

Among the key enzymes for Phase II of the detoxification process are members of the glutathione S-transferase (GST) superfamily [3]. GSTs are typically small proteins (200–250 amino acids) that are activated in response to oxidative damage and/or exposure to a large variety of toxins [4]. GSTs catalyze the conjugation of reduced glutathione (GSH) to hydrophobic xenobiotics, such as naturally occurring toxins and anthropogenically derived pharmaceuticals and pesticides [5]. The coupling of the xenobiotic to GSH increases the solubility of the toxin, thus facilitating its excretion [5].

The GSTs are a highly diverse protein superfamily, but can be divided into three distinct classes based on their cellular location, *i*.*e*., cytosolic, mitochondrial and microsomal [4]. The cytosolic class, which is primarily involved in cellular detoxification [6], contains seven subclasses (Delta, Epsilon, Omega, Sigma, Theta, Mu and Zeta). Six subclasses are found in the insects, which lack members in the subclass Mu [6]. The cytosolic GSTs are all dimeric proteins (homo- or heterodimers) with both subunits originating from the same GST subclass [7]. Each monomer contains an amino (N)-terminal α/β-domain and a carboxyl (C)-terminal α-helical domain [6]. In all subclasses, the active site, located between the two domains, is composed of two binding sites: the highly conserved G site, which binds reduced GSH, and the highly variable H site [6]. The variability in the H-site allows GSTs to detoxify a variety of “hydrophobic” substrates [8]. The catalytic activity of a mature GST is maintained by its dimeric structure, and there is no evidence of any active monomers, which is probably due to structural differences in the G-site between the monomer and the dimer [9,10]. Members of the Delta and Epsilon subclasses have been implicated in resistance to pesticides, *e*.*g*., organophosphates, organochlorines and pyrethroids [11], while the Omega, Theta and Zeta sub-groups appear to be involved in other cellular processes, including protection against oxidative stress [12].

The mitochondrial GSTs, also referred to as Kappa GSTs, are homodimers with a single conserved thioredoxin domain [3,8]. This functional motif is similar to the N-terminal domain of the cytosolic GSTs, suggesting that these proteins may have similar substrate specificity [8]. Kappa GSTs are widely distributed in nature but are absent in insects [13]. In crustaceans, Kappa GSTs have been predicted from either genomic or transcriptomic sequence data in the daphnid *Daphnia pulex* [14] and the copepods *Tigriopus japonicus* [15], *Paracyclopina nana* [16], *Lepeophtheirus salmonis* (**Accession No.** **ACO11809**), *Caligus clemensi* (**Accession No.** **ACO15728**) and *Caligus rogercresseyi* (**Accession No.** **ACO10845**).

The microsomal GSTs are membrane-associated proteins, primarily localized to the mitochondrion and endoplasmic reticulum (ER), and are involved in eicosanoid and glutathione metabolism [3,17,18]. This class of GSTs has a single conserved domain, the membrane-associated protein in eicosanoid and glutathione metabolism (MAPEG) domain, which shares high amino acid similarity with the active sites of 5-lipoxygenase-activating protein and leukotriene-C4 synthase, suggesting that they are more distantly related to the cytosolic and mitochondrial GSTs, and may have multiple enzymatic roles that are not exclusively associated with the detoxification response [17,18].

Increases in the frequency and magnitude of toxic algal blooms and anthropogenic pollution of marine environments can have devastating impacts on the economies of coastal communities due to the resulting degradation of ecosystems, declines in marine fisheries, and negative impacts on tourism and recreational activities [19,20]. Although mitigation of the effects of xenobiotics is a high priority, effective management requires an understanding of how toxins and pollutants are transferred through the food chain [19]. Planktonic copepods are known to play a crucial role in secondary production, potentially serving as vectors in the transfer of toxins to higher trophic levels in marine food webs [21]. Alternatively, through biological processes such as detoxification, excretion and fecal pellet production, copepods may be involved in the removal of xenobiotics from ecosystems [22]. Recently, several investigations have focused on how copepods respond to toxins [23]. In the calanoid copepods *Calanus finmarchicus* and *Calanus helgolandicus*, GSTs have been used as biomarkers of the detoxification response to both natural toxins (phytoplankton toxins) and anthropogenic pollutants [24–27]. Because of limited genomic resources, these studies have depended on single GSTs as biomarkers [24–28]. However, given the multiplicity and high diversification of the GST superfamily, these studies may not fully represent the copepods’ physiological response to a xenobiotic. Thus, to understand the role of the GSTs in detoxification in marine crustaceans, this protein superfamily must be better characterized. Genomic data from insects, including *Drosophila melanogaster* and *Anopheles gambiae* [11], suggest the presence of 30 or more genes in the GST superfamily. Using insect proteins as queries, just 12 GSTs were identified in the transcriptome of the intertidal copepod *T*. *japonicus* ([15], Roncalli, unpublished). The identification of only a small number of GSTs in *T*. *japonicus* raises the question as to whether copepods may exhibit lower GST diversity than insects.

*C*. *finmarchicus*, one of the most abundant mesozooplankton species in the North Atlantic Ocean [29–31], is consumed by many economically-important fishes such as cod, mackerel and herring [32,33]. Thus, *C*. *finmarchicus* has been the focus of many ecological studies in the Gulf of Maine, which is well known for frequent blooms of the toxic dinoflagellate, *Alexandrium fundyense* [22]. Recently, a *de novo* reference transcriptome was assembled for *C*. *finmarchicus* from the Gulf of Maine that included transcripts for six developmental stages [34]. It has been estimated that this transcriptome, which was assembled from over 400 million reads (paired end, 100 bp), includes at least 65% of the complete set of *C*. *finmarchicus* transcripts [34]. This estimate was confirmed by other studies that used the transcriptome to characterize neural signaling molecules in this crustacean [35–38]. Here, this transcriptome was mined for putative GST-encoding transcripts. These data were compared to a second *de novo* transcriptome, generated independently from individuals from a single stage (pre-adult) and originating from the Norwegian Sea [39]. Using known GST protein sequences from insects and other crustaceans as input queries, multiple putative GSTs belonging to the cytosolic, mitochondrial and microsomal classes were identified and characterized from this species. Comparison of the deduced *C*. *finmarchicus* GSTs with those from the insect *D*. *melanogaster* and the crustaceans *D*. *pulex* and *T*. *japonicus* established that *C*. *finmarchicus* GST complexity is comparable to those of insects, with the individual proteins showing similarities to both those of insects and of crustaceans. In addition, the relative expression of the putative GST-encoding transcripts was assessed across development. While the relative expression of members of the microsomal and mitochondrial classes was similar in naupliar and copepodite stages, those belonging to several cytosolic subclasses showed low expression in embryos, intermediate expression in early life stages (naupliar and early copepodite stages), and high expression in the pre-adult (late copepodite, CV) and adult stages. Gene diversity was highest for the cytosolic GSTs, specifically in the Delta and Sigma subclasses. These findings are consistent with this gene superfamily playing a critical role in the copepods’ physiological response to environmental stressors, and they lay the foundation for future studies on the function of GSTs in *C*. *finmarchicus* and other copepods.

## Materials and Methods

### *Calanus finmarchicus* transcriptome

Initial searches for *C*. *finmarchicus* GST-encoding transcripts were performed on the *de novo* assembled transcriptome obtained from animals from the Gulf of Maine; a detailed description of the generation, quality and coverage of this transcriptome can be found in Lenz et al. [34]. Briefly, multiplexed gene libraries were generated from RNA collected from six developmental stages: embryo, early nauplius (NI-NII), late nauplius (NV-NVI), early copepodite (CI-CII), late copepodite (CV) and adult female (CVI). Library sequencing was performed using the Illumina HiSeq 2000 platform, generating 415 million, paired-end raw reads (100 base pair long) from the combined samples. These reads were *de novo* assembled using Trinity software generating a total of 206,041 unique transcripts (contigs). The assembled transcripts were submitted to the National Center of Biotechnology Information (NCBI; www.ncbi.nlm.nih.gov) and can be accessed via **Bioproject** **PRJNA236528** [34].

### *In silico* transcriptome mining

Searches of the *C*. *finmarchicus de novo* assembly for putative GST-encoding transcripts were conducted using the tera-tblastn algorithm of DeCypher Tera-BLASTP on a TimeLogic DeCypher server; detailed descriptions of the search method are provided in Christie et al. [35–38] and Lenz et al. [34]. Known GST proteins, the majority from the copepod *T*. *japonicus* [15], were used as the query sequences for all tera-tblastn searches. GST proteins from the insect *D*. *melanogaster* and the daphnid *D*. *pulex* were used as queries to search for the cytosolic Epsilon GST subclass (insect specific [5]) and the mitochondrial Kappa class, respectively. Lastly, the nucleotide sequences of five *C*. *finmarchicus* expressed sequence tags (ESTs) previously identified as encoding putative GSTs [40] were used as queries to search the *de novo* transcriptome using the tera-tblastx algorithm. The default parameters of both tera-tblastn and tera-tblastx were used for all searches.

### Protein vetting via reciprocal BLAST and structural motif analyses

To confirm that the putative proteins reported here are true members of the GST superfamily, each was subjected to a well-established vetting protocol that involved both reciprocal BLAST and structural motif analyses; this workflow is described in detail in recent publications [34–38]. In brief, each of the *C*. *finmarchicus* transcripts identified as encoding a putative GST was fully translated using the ‘‘Translate” tool of ExPASy (http://web.expasy.org/translate/) and then the deduced protein used as the input query for a blastp search of the non-redundant arthropod protein sequences (excluding *C*. *finmarchicus* proteins) curated at NCBI (http://blast.ncbi.nlm.nih.gov/Blast.cgi). Each deduced protein was then aligned with its top blastp protein hit using MAFFT version 7 [41–43], and amino acid identity/similarity between the sequences was calculated. Percent identity between two proteins was defined as the number of identical amino acids present in the alignment (represented by ‘‘*” in the MAFFT output) divided by the total number of amino acids in the longest sequence (x100). Amino acid similarity was defined as the number of identical and similar amino acids (the latter represented by the ‘‘:” and ‘‘.” symbols in the protein alignment) divided by the total number of amino acids in the longest sequence (x100). In the case of partial proteins, amino acid identity and similarity were calculated as described above, but only for the region of overlap.

Protein structural motifs were analyzed using the online program SMART (http://smart.embl-heidelberg.de/) [44,45]. Proteins were screened to confirm that each possessed the complement of structural domains expected for members of their respective GST class/subclass. In all figures showing protein sequences, the functional domains have been highlighted using a common color-coding: GST N-terminal domain, black; GST C-terminal domain, red; microsomal MAPEG domain, green. Proteins described as ‘‘full-length” are ones that possessed a stop codon at the 5’ end prior to the first “start” methionine and are flanked on the 3’ end by a second stop codon (or have a “start” methionine that matched the position of the initial “start” methionine in the protein query used for its identification). Proteins described as ‘‘partial” lacked a start methionine (referred to here as C-terminal partial proteins), a stop codon (referred to here as N-terminal partial proteins), or both of these features (referred to here as an internal protein fragment).

### Comparison of *Calanus finmarchicus* GST diversity with that of selected insect/crustacean species

The collection of GSTs predicted from *C*. *finmarchicus* was compared to those from the fruit fly *D*. *melanogaster* [46] and the crustaceans *D*. *pulex* [14] and *T*. *japonicus* [15]. It should be noted, that the proteins available for *T*. *japonicus* GSTs were derived from transcribed sequences, whereas those from both *D*. *melanogaster* and *D*. *pulex* were obtained from genomic data. Thus, the collection obtained for *T*. *japonicus* may be an incomplete set of GST proteins as not all may have been transcribed at the time of mRNA isolation; while those reported for *D*. *melanogaster* and *D*. *pulex* may contain ones that are not actually transcribed in the species in question.

Phylogenetic analysis was performed for GST members of the cytosolic class identified in *C*. *finmarchicus* and the cytosolic GSTs from the insect *D*. *melanogaster* and the crustaceans *D*. *pulex* and *T*. *japonicus*. Phylogenetic trees of the cytosolic GSTs were used to establish the relationship among the subclasses in insects [5,46]. Here, the phylogenetic tree was used to support the assignment of predicted GST proteins into subclasses and to establish their relationship to each other and to those from *D*. *melanogaster*, *D*. *pulex* and *T*. *japonicus*. For the construction of an unrooted phylogenetic tree, the publicly available cytosolic GST protein sequences for *D*. *melanogaster*, *D*. *pulex* and *T*. *japonicus* were downloaded from NCBI using the GenBank accession numbers listed in previous publications [14,15,46]. In addition, for the completeness of the *D*. *pulex* dataset, GST proteins were also searched for by name (“glutathione S-transferase”) and extracted from the genome assembly (daphnia_genes2010_beta3.aa.gz) accessible via wFleaBase (http://wfleabase.org/). Amino acid sequences for GST proteins were aligned using MAFFT software [41–43], and resultant alignments were trimmed and corrected manually to remove non-conserved regions and obvious alignment errors. The best-fit likelihood model for each alignment was determined using ProtTest [47]. Phylogenetic reconstruction was performed with MrBayes 3.2 [48] with four independent runs of four chains each and 10,000,000 generations, using the WAG substitution model of protein evolution [49] and a gamma distribution of rates with four categories. A consensus tree was obtained by discarding the initial 2,500,000 generations as burn-in. Maximum likelihood bootstrap analysis was performed with RAxML 8 [50], with 1,000 bootstrap replicates using the WAG substitution model of protein evolution and a gamma distribution of rates. The unrooted consensus tree from MrBayes was visualized in FigTree v1.3.1 (http://www.tree.bio.ed.ac.uk/software/figtree/) with bootstrap values >50% reported.

### Expression of GSTs during development

The relative expression of the identified *C*. *finmarchicus* GSTs was examined across developmental stages (embryo, early nauplius, late nauplius, early copepodite, late copepodite and adult female) as described in earlier publications [34–38]. In brief, Illumina reads for six developmental stages obtained in either 2011 [34] or 2012 [38] were mapped against each of the identified *C*. *finmarchicus* nucleotide sequences using Bowtie software (version 2.0.6; with a setting of 2 mismatches) [51]. Prior to the mapping step, reads were quality filtered using FASTX Toolkit software (version 0.013; http://hannonlab.cshl.edu/fastx_toolkit), with a Phred quality score of 20 used as the acceptance cutoff (*i*.*e*. low quality reads were removed from each dataset). Relative expression was computed for each transcript as reads per kilobase transcript per million reads (RPKM) using a custom written Perl script. Briefly, the total number of reads mapped to each transcript was divided by the total number of mapped reads to the reference transcriptome multiplied by the length of the transcript [52].

### Comparison between two *C*. *finmarchicus de novo* transcriptomes

In addition to the transcriptome generated from animals obtained from the Gulf of Maine [34], a second *de novo* transcriptome was independently generated by Tarrant and colleagues using material obtained from pre-adult (stage CV) *C*. *finmarchicus* and publicly deposited [39]. For this transcriptome, total RNA was extracted from individuals collected from both surface waters in Trondheim fjord (Norwegian Sea) and from individuals reared in culture, gene libraries were prepared and sequenced on the Illumina platform (**Bioproject No.** **PRJNA2311645**). The”Norwegian Sea” transcriptome was mined for GST-encoding transcripts using the proteins deduced from the “Gulf of Maine” transcriptome and the *T*. *japonicus* GSTs as queries. The goal here was to verify the diversity of the putative GSTs in *C*. *finmarchicus* using a *de novo* transcriptome that had been generated independently, and to compare the predicted GSTs from the two populations. For these BLAST analyses, the searched database of the online program tblastn (National Center for Biotechnology Information, Bethesda, MD; http://blast.ncbi.nlm.nih.gov/Blast.cgi) was set to ‘‘Transcriptome Shotgun Assembly (TSA)” and restricted to sequence data from the ‘‘*Calanus finmarchicus* (taxid: 6837)”, which allowed access to the Norwegian Sea dataset. All hits returned by a given search were translated into proteins and checked manually for homology to the target query as described earlier. Comparisons between sequences included aligning the predicted proteins with their query and determining their percent amino acid identity. When the translated proteins differed in length, percent amino acid identity was determined only for the region of overlap.

## Results

### Mining of a *Calanus finmarchicus de novo* transcriptome for transcripts encoding glutathione S-transferase proteins

A total of 39 putative GST-encoding transcripts were retrieved from the Gulf of Maine *C*. *finmarchicus* transcriptome using known GSTs from the crustaceans *T*. *japonicus* (a copepod) and *D*. *pulex* (a cladoceran) and the insect *D*. *melanogaster* as queries (Table 1). The putative GST-encoding transcripts identified from *C*. *finmarchicus* included representatives of all three classes, *i*.*e*., cytosolic, mitochondrial and microsomal, with the majority encoding putative members of the cytosolic class (32 transcripts) in six subclasses (Delta, Theta, Mu, Omega, Sigma and Zeta). Transcripts encoding six microsomal GSTs (subclasses 1 and 3) and one mitochondrial (Kappa) GST were also identified (Table 1). Interestingly, the searches using cytosolic GST Delta, Theta and Epsilon subclass members as queries yielded identical sets of *C*. *finmarchicus* sequences (11 transcripts in total; Table 1). Using Delta and Theta GSTs from the copepod *T*. *japonicus* as queries, the BLAST-generated E-values for the eleven putative GST-encoding transcripts overlapped extensively and ranged from 10^–68^ to 10^–9^. Not surprisingly, the BLAST-generated E-values for the same list of transcripts were higher using an insect-specific Epsilon GST from *D*. *melanogaster* as a query, and ranged between 10^–36^ and 10^–9^. As will be presented later, reciprocal protein BLAST and phylogenetic analyses were used to resolve this apparent conundrum.

**Table 1:** Search results from *in silico* mining of a *de novo* transcriptome from *Calanus finmarchicus* using glutathione S-transferase (GST) queries obtained from the copepod *Tigriopus japonicus*, the cladoceran *Daphnia pulex* and the insect *Drosophila melanogaster*.

Class	Subclass	Transcript accession number	Transcript length†
Cytosolic	Delta/Theta/Epsilon*	GAXK01204954	888
		GAXK01204965	921
		GAXK01204950	852
		GAXK01204940	965
		GAXK01204947	1182
		GAXK01204957	902
		**GAXK01204968**	764
		GAXK01204953	991
		GAXK01073468	401
		GAXK01096295	914
		GAXK01035521	823
	Mu	GAXK01204944	957
		GAXK01204956	741
		GAXK01204948	742
		GAXK01204952	1686
		GAXK01204958	785
	Omega	GAXK01204960	984
		GAXK01016325	1410
		GAXK01164502	894
	Sigma	**GAXK01204949**	1346
		**GAXK01204945**	839
		GAXK01204943	815
		GAXK01204951	733
		GAXK01204959	955
		GAXK01204946	1022
		GAXK01204964	771
		GAXK01204961	884
		GAXK01204942	756
		GAXK01204966	881
	Zeta	GAXK01204939	1033
		GAXK01204941	2790
		GAXK01084871	359
Mitochondrial	Kappa	GAXK01046934	1108
Microsomal	mGST-1	GAXK01178264	771
		GAXK01081966	347
	mGST-3	GAXK01204963	54
		GAXK01204967	465
		GAXK01204962	7866
		**GAXK01204955**	680

Transcript lengths (base pairs) are given for each transcript identified.

†Length in nucleotides.

Accession Nos for query proteins: GST Delta *T*. *japonicus* (ACE81244, ACE81245). GST Theta *T*. *japonicus* (ACE81253). GST Epsilon *D*. *melanogaster* (AAF57701). GST Mu *T*. *japonicus* (ACE81251, ACE81252, ACE81254). GST Omega *T*. *japonicus* (ACE81246). GST Sigma *T*. *japonicus* (AAY89316). GST Zeta *T*. *japonicus* (ACE81250). Mitochondrial GST Kappa *D*. *pulex* (EFX86155). Microsomal GST-1 *T*. *japonicus* (ACE81248). Microsomal GST-3 *T*. *japonicus* (ACE81249) as listed.

*Queries for Delta, Theta and Epsilon subclasses deduced the identical list of 11 transcripts.

*C*. *finmarchicus* transcripts whose accession numbers are shown in bold font have sequence support from expressed sequence tag data (see text).

It should be noted that the EST database for *C*. *finmarchicus* contains five sequences annotated as GSTs. Tblastx analysis showed that two of these ESTs (**Accession Nos.** **ES387233** **and** **FG632831**) matched two of the putative cytosolic Sigma GSTs identified here, with another (**Accession No.** **FK671334**) matching one of the cytosolic Delta sequences (see below), and a fourth (**Accession No.** **ES387262**) matching a microsomal GST, with amino acid identity >90% for each of the respective pairs (in bold in Table 1). The fifth EST annotated as a GST (**Accession No.** **ES387185**) did not generate significant hits from the *Calanus* transcriptome, and a subsequent blastp search of the non-redundant protein database suggests that the protein encoded by this EST may not be a GST. The predicted protein is only 43 amino acids long, and while it is most similar to the C-terminus of a GST from the nematode *Caenorhabditis brenneri* (**Accession No.** **EGT40878**), the E-value is very high (10^–4^).

Class and subclass assignments of the GSTs in Table 1 were confirmed by translating each sequence into a predicted protein, followed by reciprocal BLAST and structural analyses.

### Cytosolic GSTs

#### Delta, Epsilon and Theta subclasses

**Fig 1:**
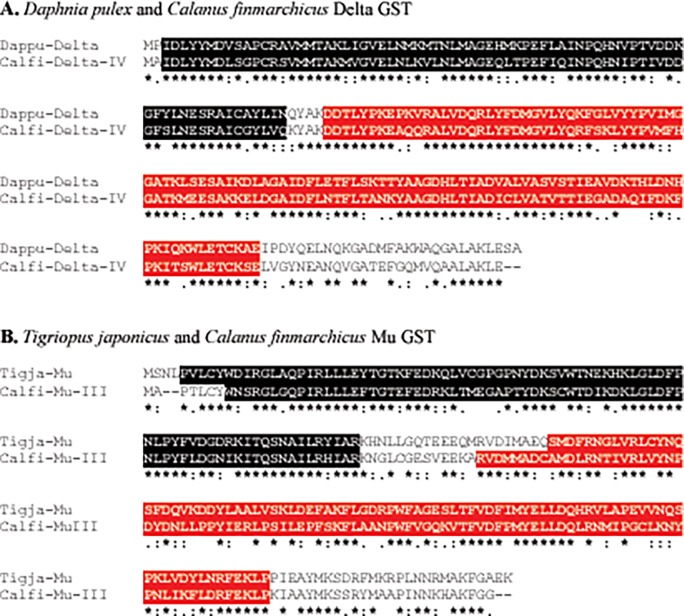
Alignment of selected *Calanus finmarchicus* Delta GST (Calfi-Delta-IV) and Mu GST (Calfi-Mu-III) proteins with their top arthropod protein BLAST hits. (A) Alignment of *D*. *pulex* Delta GST (Dappu-Delta) (**Accession No.** **EFX81633**; 222 amino acids long) and Calfi-Delta-IV (220 amino acids long). (B) Alignment of the *T*. *japonicus* Mu GST (Tigja-Mu; **Accession No.** **ACE81254**; 221 amino acids long) and Calfi-Mu-III (222 amino acids long). In each panel, ‘‘*” located beneath the alignment indicates residues that are identical in the two sequences, while ‘‘:” and ‘‘.” indicate conservatively substituted (similar) amino acids shared between the protein pairs. Amino acids highlighted in black are the ones predicted by SMART analysis to form the conserved amino (N)-terminal domain (GSTN), amino acids highlighted in red represent the conserved carboxyl (C)-terminal domain (GSTC).

Eight full-length and three partial proteins were predicted from the 11 transcripts putatively identified in the original searches as belonging to either the Delta, Theta or Epsilon GST subclass (Table 2). Structural analysis confirmed the presence of GST N-terminal and GST C-terminal domains in all of the predicted full-length proteins. The three partial proteins possessed the expected complement of domains consistent with their incomplete nature (Table 2 and Fig 1A).

**Table 2:** Annotation of putative glutathione S-transferase-encoding transcripts from Table 1, using reciprocal BLAST results and protein domain analysis.

Class—subclass and assigned protein name	Transcript accession No.	Deduced protein length†	Structural domains	Species	Protein accession No.	E-value	% amino acid identity/similarity
**Cytosolic—Delta**							
Calfi-Delta-I	GAXK01204953	217*	GSTN, GSTC	*Lepeophtheirus salmonis*	ACO12967	8.9e-44	40/78
Calfi-Delta-II	GAXK01204968	217**	GSTN, GSTC	*Lepeophtheirus salmonis*	ACO12967	8.9e-36	43/77
Calfi-Delta-III	GAXK01204940	218*	GSTN, GSTC	*Lepeophtheirus salmonis*	ACO12967	7.0e-33	39/80
Calfi-Delta-IV	GAXK01204965	220**	GSTN, GSTC	*Daphnia pulex*	EFX81633	2.8e-29	69/70
Calfi-Delta-V	GAXK01204954	221*	GSTN, GSTC	*Tigriopus japonicus*	ACE81245	2.8e-29	57/86
Calfi-Delta-VI	GAXK01204957	237**	GSTN, GSTC	*Lepeophtheirus salmonis*	ACO12967	1.1e-28	38/80
Calfi-Delta-VII	GAXK01204947	262*	GSTN, GSTC	*Lepeophtheirus salmonis*	ADD38060	3.2e-25	59/89
Calfi-Delta-VIII	GAXK01073468	113***	GSTN	*Tigriopus japonicus*	ACE81245	7.5e-27	33/44
Calfi-Delta-IX	GAXK01204950	178***	GSTC	*Caligus clemensi*	ACO15541	3.2e-17	44/68
Calfi-Delta-X	GAXK01035521	201*	GSTN, GSTC	*Caligus rogercresseyi*	ACO15749	2.2e-10	21/54
Calfi-Delta-XI	GAXK01204939	330**	GSTN, GSTC	*Lepeophtheirus salmonis*	ADD38823	1.3e-07	27/54
**Cytosolic—Theta**							
Calfi-Theta-I	GAXK01096295	262**	GSTN, GSTC	*Locusta migratoria*	AEB91980	1.3e-79	45/72
**Cytosolic—Mu**							
Calfi-Mu-I	GAXK01204944	222*	GSTN, GSTC	*Caligus clemensi*	ACO15225	2.2e-105	65/89
Calfi-Mu-II	GAXK01204956	222**	GSTN, GSTC	*Macrobrachium nipponense*	AGJ70295	3.1e-95	65/85
Calfi-Mu-III	GAXK01204948	222**	GSTN, GSTC	*Tigriopus japonicus*	ACE81254	1.0e-90	48/69
Calfi-Mu-IV	GAXK01204952	222*	GSTN, GSTC	*Tigriopus japonicus*	ACE81254	9.5e-90	58/82
Calfi-Mu-V	GAXK01204958	222*	GSTN, GSTC	*Tigriopus japonicus*	ACE81254	4.1e-59	45/76
**Cytosolic—Omega**							
Calfi-Omega-I	GAXK01204960	268*	GSTN, GSTC	*Riptortus pedestris*	BAN21163	5.3e-46	36/64
Calfi-Omega-II	GAXK01164502	235***	GSTN	*Acromyrmex echinatior*	EGI63780	3.2e-32	35/64
Calfi-Omega-III	GAXK01016325	272***	GSTN	*Coptotermes formosanus*	AFZ78680	2.6e-51	36/68
**Cytosolic—Sigma**							
Calfi-Sigma-I	GAXK01204964	200**	GSTN, GSTC	*Daphnia pulex*	EFX82687	3.1e-44	36/70
Calfi-Sigma-II	GAXK01204942	204*	GSTN, GSTC	*Daphnia pulex*	EFX82672	2.0e-42	40/67
Calfi-Sigma-III	GAXK01204943	216**	GSTN, GSTC	*Tigriopus japonicus*	AAY89316	7.2e-45	40/74
Calfi-Sigma-VI	GAXK01204959	217*	GSTN, GSTC	*Apis florae*	XP_003694330	8.2e-38	35/71
Calfi-Sigma-V	GAXK01204951	218*	GSTN, GSTC	*Tigriopus japonicus*	AAY89316	5.0e-33	38/70
Calfi-Sigma-VI	GAXK01204961	218****	GSTN, GSTC	*Daphnia pulex*	EFX82672	5.1e-39	38/69
Calfi-Sigma-VII	GAXK01204946	225*	GSTN, GSTC	*Daphnia pulex*	EFX63772	2.1e-39	38/76
Calfi-Sigma-VIII	GAXK01204966	239*	GSTN, GSTC	*Daphnia pulex*	EFX82672	1.8e-62	45/75
Calfi-Sigma-XI	GAXK01204949	196*	GSTN, GSTC	*Folsomia candida*	AGZ95070	1.2e-35	35/51
Calfi-Sigma-II	GAXK01204945	223*	GSTN, GSTC	*Megachile rotundata*	XP_00370954	1.1e-37	36/56
**Cytosolic—Zeta**							
Calfi-Zeta-I	GAXK01204941	225**	GSTN, GSTC	*Drosophila melanogaster*	NP_731358	5.0e-84	54/82
Calfi-Zeta-II	GAXK01084871	113***	GSTC	*Bactrocera dorsalis*	AFI99067	6.1e-27	22/32
**Mitochondrial—Kappa**							
Calfi-Kappa-I	GAXK01046934	256*	THX	*Paracyclopina nana*	ADV59554	1.2e-65	36/61
**Microsomal—mGST-1**							
Calfi-mGST-1-I	GAXK01081966	93***	MAPEG	*Drosophila persimilis*	XP_002023020	4.0e-42	36/61
Calfi-mGST-1-II	GAXK01081966	93***	MAPEG	*Ceratitis capitata*	XP_00453691	3.2e-26	31/52
**Microsomal—mGST-3**							
Calfi-mGST-3-I	GAXK01204963	156**	MAPEG	*Acartia pacifica*	AGN29624	3.2e-60	65/84
Calfi-mGST-3-II	GAXK01204967	156**	MAPEG	*Daphnia pulex*	EFX85348	7.1e-37	46/77
Calfi-mGST-3-III	GAXK01204955	264**	MAPEG	*Daphnia pulex*	EFX85347	2.2e-34	42/73
Calfi-mGST-3-IV	GAXK01204962	145****	MAPEG	*Acartia pacifica*	AGN29624	5.1e-49	61/75

BLAST searches were limited to NCBI non-redundant protein database for arthropods (taxid: 6656).

†Length in amino acids.

* Predicted full-length protein flanked by “stop” codons at both N- and C-terminals.

** Putative full-length protein flanked by a “methionine” at the N-terminal, and a “stop” codon at the C-terminal. Identification of full-length is based on presence of expected structural domains and similarity to full-length proteins.

*** Partial protein with either the N-terminal “methionine” or C-terminal “stop” codon missing.

**** Protein originally identified as full-length, but prediction corrected to partial after alignment with their most similar transcript in the Norwegian Sea transcriptome (see text).

Abbreviations: GSTN, GST amino (N)-terminal domain; GSTC, GST carboxyl (C)-terminal domain; THX, thioredoxin-like domain; MAPEG, membrane-associated proteins in eicosanoid and glutathione metabolism domain.

Reciprocal BLAST analysis identified ten of the cytosolic GSTs as members of the Delta subclass, with nine of these putative proteins returning Delta GSTs from other copepod species as the top BLAST hit. With respect to these proteins, five, Calfi-Delta-I, Calfi-Delta-II, Calfi-Delta-III, Calfi-Delta-VI and Calfi-Delta-VII, were found to be most similar to Delta GSTs from *L*. *salmonis*, while two, Calfi-Delta-V and Calfi-Delta-VIII, were most similar to a Delta GST from *T*. *japonicus* (Table 2). The tenth protein, Calfi-Delta-IV, was identified as most similar to a Delta GST from the cladoceran *D*. *pulex* (Table 2). The reciprocal BLAST of the eleventh protein in the GST Delta/Theta/Epsilon list (Table 1) identified it as a GST in the Theta subclass, being most similar to a Theta GST protein of the insect *Locusta migratoria* (Table 2). None of the 11 GSTs resulting from the Delta/Theta/Epsilon searches (see above) were found to be members of the Epsilon subclass, which is consistent with the hypothesis that this subclass is insect-specific [53].

Interestingly, a GST initially identified via transcriptome mining as a cytosolic Zeta subclass GST (**Accession No.** **GAXK01204939**) was ultimately determined via reciprocal BLAST analysis to be a member of the Delta subclass; the top hit returned for this protein, Calfi-Delta-XI, was a cytosolic Delta GST from the copepod *L*. *salmonis* (Table 2).

Alignments of each *C*. *finmarchicus* putative Delta GST with its respective top BLAST hit showed 21%-69% amino acid identity and 54%-89% amino acid similarity for the full-length proteins (Table 2 and Fig 1A). Alignments of the regions of overlap between the partial *C*. *finmarchicus* sequences and their top BLAST hits revealed 33%-44% identity and 44%-68% similarity in amino acid sequence (Table 2 and Fig 1A). Similarly, alignment of Calfi-Theta-I with its top protein hit showed 45% amino acid identity and 72% amino acid similarity between the two proteins (Table 2). Pairwise alignments of four *C*. *finmarchicus* Delta GSTs (Calfi-Delta-I, Calfi-Delta-II, Calfi-Delta-III and Calfi-Delta-VI) that had the identical top hit from the copepod *L*. *salmonis* (Table 2) showed that these predicted proteins shared only 27%-50% amino acid identity with each other. Likewise, alignment of Calfi-Delta-V and Calfi-Delta-VIII, which both shared the same *T*. *japonicus* Delta GST as their top protein hits, showed only 33% amino acid identity between the two proteins. The large differences in amino acid sequence among these *C*. *finmarchicus* Delta subclass GSTs are consistent with the Trinity software assembly results that placed the transcripts that encode them into unique “comps” which represent transcripts encoded by different genes [54], a finding that is consistent with the multiplicity of insect GST genes.

#### Mu subclass

Five full-length proteins were predicted from the five transcripts identified in the original search as encoding putative members of the Mu subclass (Table 2). Each of these proteins possesses the conserved GST N-terminal and GST C-terminal domains (Table 2 and Fig 1B). Reciprocal BLAST analysis confirmed the five proteins as members of the Mu subclass, with four of the proteins returning as top BLAST hits Mu GSTs from other copepod species, and one, Calfi-Mu-II, a Mu GST from the river prawn, a decapod crustacean (Table 2). Three of these proteins (Calfi-Mu-III, Calfi-Mu-VI and Calfi-Mu-V) were found to be most similar to the *T*. *japonicus* Mu GST that was used in the initial search of the transcriptome (Table 2).

Alignments of each of the putative *C*. *finmarchicus* Mu GSTs with its respective top hit revealed 45%-65% amino acid identity and 69%-89% amino acid similarity between the protein pairs (Table 2). Pairwise alignments of the three Mu GSTs (Calfi-Mu-III, Calfi-Mu-VI and Calfi-Mu-V) that had the identical top hit from the copepod *T*. *japonicus* (Table 2) showed that these predicted proteins shared 34%-60% amino acid identity, suggesting that different genes encode them.

#### Omega subclass

One full-length and two partial proteins were predicted from the three transcripts identified in the original search as encoding putative Omega subclass GSTs (Table 2). Structural analysis confirmed the presence of GST N-terminal and GST C-terminal domains for the full-length protein, while the two partial proteins possessed only the N-terminal domain (Table 2). Results from the reciprocal BLAST analysis identified the three predicted proteins as members of the Omega subclass, returning Omega GSTs from ants as the top BLAST hits (Table 2). Alignment of each *C*. *finmarchicus* putative Omega GST and its top BLAST hit revealed amino acid identities/similarities ranging from 35%-36% and 64%-68%, respectively (Table 2).

#### Sigma subclass

Ten full-length proteins were predicted from the 10 transcripts putatively identified in the initial search as encoding members of the Sigma subclass (Table 2). Structural analysis confirmed the presence of the GST N-terminal and GST C-terminal domains in each protein (Table 2).

Reciprocal BLAST analysis confirmed all 10 predicted proteins as members of the Sigma subclass. Seven of the *C*. *finmarchicus* proteins returned Sigma GSTs from other crustaceans as their top BLAST hits, while three returned Sigma GSTs from insects as the most similar proteins (Table 2). Specifically, five of the *C*. *finmarchicus* proteins (Calfi-Sigma-I, Calfi-Sigma-II, Calfi-Sigma-VI, Calfi-Sigma-VII and Calfi-Sigma-VIII) were found to be most similar to Sigma GSTs from *D*. *pulex*, with two (Calfi-Sigma-III and Calfi-Sigma-V) most similar to a Sigma GST from the copepod *T*. *japonicus* (Table 2). Calfi-Sigma-IV, Calfi-Sigma-IX and Calfi-Sigma-X returned Sigma GSTs from the insects *Apis florea*, *Folsomia candida* and *Megachile rotundata*, respectively, as their top BLAST hits (Table 2). Alignments of each *C*. *finmarchicus* putative Sigma GST with its respective top hit revealed 35%-45% amino acid identity and 51%-76% amino acid similarity between the protein pairs (Table 2). Pairwise alignments of the three *C*. *finmarchicus* Sigma GSTs (Calfi-Sigma-II, Calfi-Sigma-VI and Calfi-Sigma-VIII) that had the identical top BLAST hit (Table 2) showed only 36%-42% amino acid identity to each other. Likewise, alignment of Calfi-Sigma-III and Calfi-Sigma-V, both most similar to the same *T*. *japonicus* Sigma GST, showed 42% amino acid identity between the two proteins.

#### Zeta subclass

One full-length protein and two partial proteins were predicted from the three transcripts identified in the original search as putatively encoding Zeta subclass GSTs. The partial protein encoded by transcript **GAXK01204939** was found to be most similar to a Delta GST, and was assigned to the Delta subclass accordingly (Calfi-Delta-XI, see above). Structural analyses of the two remaining proteins (Calfi-Zeta-I and Calfi-Zeta-II), confirmed the presence of GST N-terminal and GST C-terminal domains in the full-length protein and the GST C-terminal domain in the partial sequence (Table 2). Reciprocal BLAST analyses identified these two proteins as members of the Zeta subclass, returning Zeta GSTs from the insects *D*. *melanogaster* and *Bactrocera dorsalis* as the top hits, respectively (Table 2). Alignment of Calfi-Zeta-I with its top hit revealed 54% amino acid identity and 82% amino acid similarity; alignment of the extant sequence of Calfi-Zeta-II and the corresponding portion of its top hit revealed 22% amino acid identity and 32% amino acid similarity (Table 2).

### Mitochondrial class

A single full-length protein was predicted from the transcript putatively identified as encoding a mitochondrial Kappa GST (Table 2). Structural analysis revealed that this protein possesses a mitochondrial GST thioredoxin-like domain, which is typical of mitochondrial GSTs (Table 2). Reciprocal BLAST analysis identified the protein as a member of the mitochondrial class, returning a mitochondrial Kappa GST from the copepod *P*. *nana* as its top BLAST hit (Table 2). Alignment of the *C*. *finmarchicus* mitochondrial Kappa GST with its top hit showed 36% amino acid identity and 61% similarity between the two proteins (Table 2).

### Microsomal class

#### mGST-1

One full-length and one partial protein were predicted from the two transcripts identified in the initial search as belonging to the microsomal GST subclass 1 (Table 2). Reciprocal BLAST analyses identified both proteins as subclass 1 microsomal GSTs, returning microsomal GST-1s from insects as the top BLAST hits (Table 2). Alignment of Calfi-mGST-1-I with its top hit revealed 36% amino acid identity/61% amino acid similarity between the two proteins; 31% amino acid identity/52% amino acid similarity was seen between the known portion of Calfi-mGST-1-II and its top hit (Table 2).

**Fig 2:**
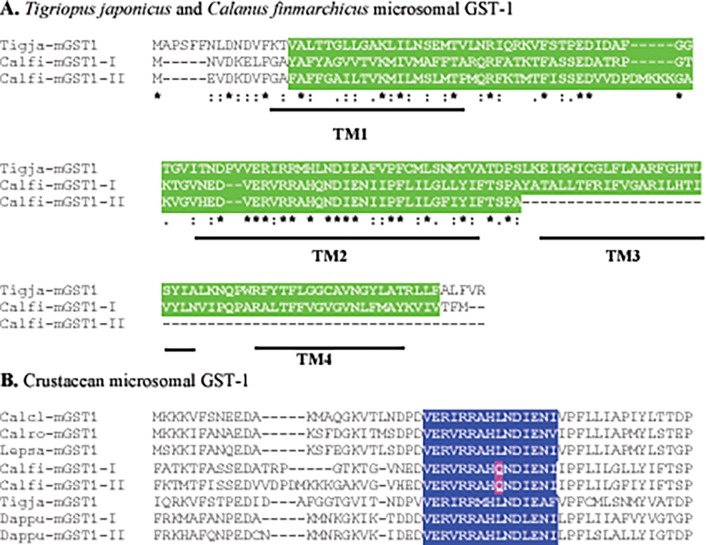
Alignment of *Calanus finmarchicus* microsomal glutathione S-transferase subclass 1 proteins with mGST-1s from other crustaceans. (A) Alignment of *C*. *finmarchicus* putative microsomal GST-1 proteins (Calfi-mGST-1-I and Calfi-mGST-1-II) with the *T*. *japonicus* query used in their discovery (Tigja-mGST-1; **Accession No.** **ACE81248**). Highlighted in green are amino acids in the conserved MAPEG structural domain identified using SMART software. The abbreviation “TM” indicates predicted transmembrane regions in the *C*. *finmarchicus* mGST-1 proteins. The ‘‘*” located beneath each alignment indicates residues that are identical in the two sequences, while ‘‘:” and ‘‘.” indicate conservatively substituted (similar) amino acids shared between the protein pairs. (B) Multiple alignments of *C*. *finmarchicus* microsomal GST-1 proteins (Calfi-mGST-1-I and Calfi-mGST-1-II) with publicly available mGST-1s from the crustaceans *C*. *clemensi* (Calcl), *C*. *rogercresseyi* (Calro), *L*. *salmonis* (Lepsa), *T*. *japonicus* (Tigja) and *D*. *pulex* (Dappu). The conserved motif consisting of 16 amino acids (VERVRR*X*HLND*X*ENI*X*, where the three *X*s represent variable residues) is highlighted in blue. The non-conservative substitution found only in *C*. *finmarchicus* is highlighted in pink.

Structural analysis identified a single MAPEG domain with the typical four transmembrane regions in both *C*. *finmarchicus* mGST-1 proteins (Table 2 and Fig 2A). Within the conserved MAPEG region, microsomal GST-1 proteins are characterized by an amino acid pattern that is shared by both arthropods and vertebrates [17,53,55,56]. The pattern consists of a highly conserved motif of 16 amino acids (VERVRR*X*HLND*X*ENI*X*) where the three *X*s represent variable amino acids [17]. The *C*. *finmarchicus* microsomal GST-1 proteins identified here (Calfi-mGST-1-I and Calfi-mGST-1-II) were aligned with mGST-1 amino acid sequences from other crustaceans, specifically the copepods *C*. *clemensi*, *C*. *rogercresseyi*, *L*. *salmonis* and *T*. *japonicus*, and the cladoceran *D*. *pulex* (Fig 2B). This alignment showed that the 16 amino acids motif VERVRR*X*HLND*X*ENI*X* was conserved in all crustaceans except for *C*. *finmarchicus*. In both *C*. *finmarchicus* sequences, there was a non-conservative substitution in the 9^th^ amino acid of the motif, specifically the stereotypical hydrophobic leucine (L) was substituted by a hydrophilic glutamine (Q) residue (Fig 2B). This amino acid substitution was also present in a protein predicted from the Norwegian Sea transcriptome (**Accession No.** **GBFB01067142**; see below). Thus, this observed amino acid substitution is unlikely to be an assembly artifact, and may be *C*. *finmarchicus*-specific (Fig 2B).

#### mGST-3

Four proteins, three full-length and one partial, were predicted from four transcripts identified as encoding putative members of microsomal GST subclass 3 (Table 2). In all four proteins, structural analysis confirmed the presence of the MAPEG domain (Table 2). Reciprocal BLAST analysis identified these proteins as microsomal GST subclass 3 members, with each protein returning a crustacean mGST-3 as its top BLAST hit (Table 2). Two of the proteins, Calfi-mGST-3-I and Calfi mGST-3-IV were found to be most similar to a mGST-3 from the copepod *Acartia pacifica*, while Calfi-mGST-3-II and Calfi-mGST-3-III were most similar to a mGST-3 from *D*. *pulex* (Table 2). The percent amino acid identity/similarity between each of the *C*. *finmarchicus* mGST-3 and its top BLAST hit was 42%-65%/73%-84% (Table 2). Alignment of the two *C*. *finmarchicus* mGST3 (Calfi-mGST-3-I and Calfi-mGST-3-IV) that shared the same top hit showed 77% of amino acid identity between the two proteins.

### Glutathione S-transferase diversity in *C*. *finmarchicus*

The identification of 39 putative *C*. *finmarchicus* GSTs from the Gulf of Maine transcriptome suggests that the gene complexity found in this copepod species is comparable to that of the insect *D*. *melanogaster* (40 GST genes) and higher than that of the crustacean *D*. *pulex* (31 GSTs genes; Table 3) [14,46]. Comparison between *C*. *finmarchicus* and *D*. *pulex* indicates that the number of genes in some subclasses, *i*.*e*., the cytosolic Sigma and Theta subclasses, as well as in the microsomal GST-1 group, is very similar (Table 3). However, the gene duplication found in the *C*. *finmarchicus* cytosolic Delta subclass is higher than that reported for *D*. *pulex*, and is identical to the complexity seen in the insect *D*. *melanogaster*, which has a total of 11 Delta GST genes (Table 3). The complexity of GSTs reported for *T*. *japonicus*, another member of the Copepoda, is lower than that found for *C*. *finmarchicus*, although this may be a function of sequencing depth, since the current *T*. *japonicus* transcriptome data are more limited.

**Table 3:** Number of genes in different classes and subclasses of the glutathione S-transferase superfamily in the crustaceans *Calanus finmarchicus*, *Tigriopus japonicus* [15] and *Daphnia pulex* [14] and the insect *Drosophila melanogaster* [53].

Class	Subclass	*C*. *finmarchicus* *	*T*. *japonicus* *	*D*. *pulex* **	*D*. *melanogaster* **
Cytosolic	Delta	11	2	4	11
	Theta	1	1	1	4
	Epsilon	0	0	0	14
	Mu	5	3	6	0
	Omega	3	1	1	4
	Sigma	10	1	10	1
	Zeta	2	1	3	2
Mitochondrial	Kappa	1	1	2	0
Microsomal	mGST-1	2	1	2	4
	mGST-3	4	1	2	0
Total GSTs		39†	12	31	40

* Proteins deduced from transcriptome data.

** Proteins deduced from genomic data.

† Predicted from the Gulf of Maine transcriptome only.

**Fig 3:**
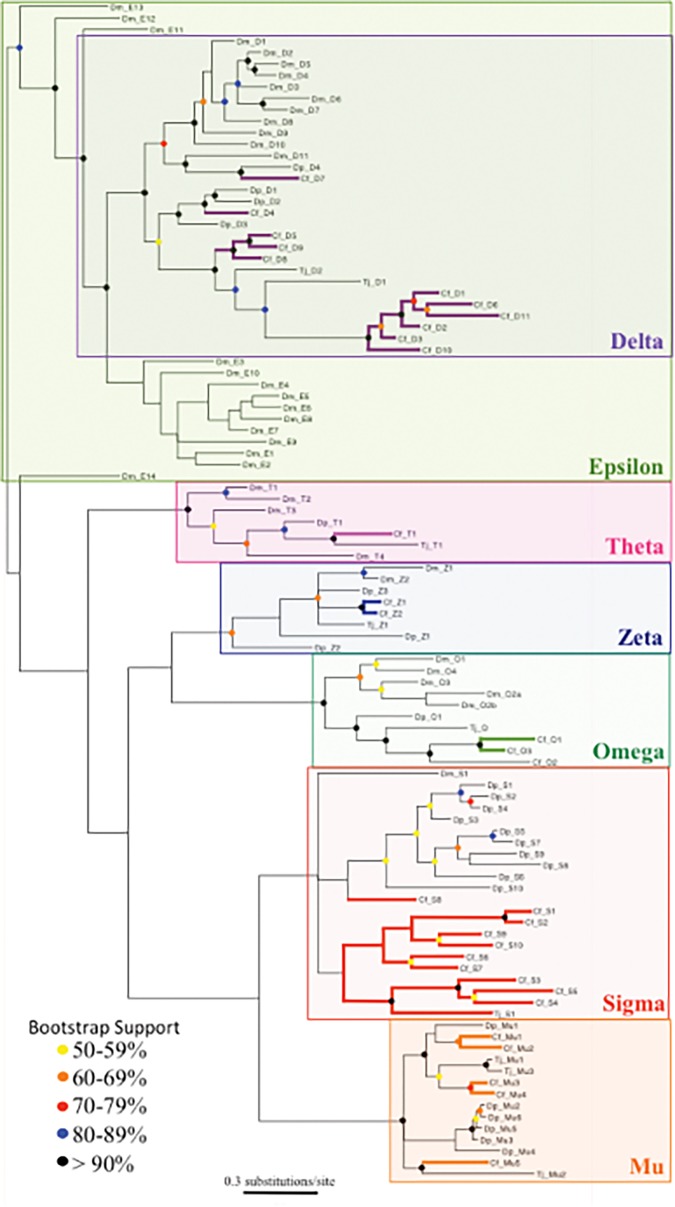
Phylogenetic tree for cytosolic GSTs from the crustacean *Calanus finmarchicus* and other selected crustacean and insect species. The consensus Bayesian likelihood tree shows the relationships between cytosolic GSTs from *C*. *finmarchicus* (Cf, in color) and those from the insect *D*. *melanogaster* (Dm), the copepod *T*. *japonicus* (Tj), and the cladoceran *D*. *pulex* (Dp). The tree was built using an analysis of 10,000,000 generations in MrBayes, excluding the initial 2,500,000 generations as burn-in. Bootstrap values were calculated using RAxML with 1,000 interactions. For 73 branches, Bayesian posterior probabilities were grater than P>0.5, 68% of those with P between 0.9 and 1 (data not shown). 73 branches had bootstrap values greater than 50% (color-coded circles).

Phylogenetic analysis based on Bayesian likelihood criteria places the deduced *C*. *finmarchicus* cytosolic GSTs (see above) into distinct clades (Fig 3), which are consistent with their classification into different subclasses. Members of the cytosolic subclasses Delta, Omega, Zeta, Mu, Sigma and Theta were identified in the phylogenetic tree with good bootstrap support (>50% for most; Fig 3). In the consensus tree, the Delta, Omega, Zeta, Mu and Theta subclasses were each recovered as monophyletic groups with bootstrap support >90% for many. The Sigma subclass was also recovered as monophyletic, with a posterior probability of P> 0.8 (data not shown), and with bootstrap support >50% for most of the branches. The Delta GSTs were recovered as monophyletic with bootstrap support >90%, but nested within the Epsilon GSTs from *D*. *melanogaster*. Despite this, none of the predicted cytosolic GSTs from *C*. *finmarchicus*, *T*. *japonicus*, or *D*. *pulex* were recovered as most closely related to individual members of the poorly resolved Epsilon subclass, consistent with this subclass being absent in these crustaceans [53].

The clustering pattern within individual subclasses varied, but in many cases all, or at least a large subset, of the *C*. *finmarchicus* GSTs within a subclass were located on a single branch. For example, in the Delta clade with 11 *C*. *finmarchicus* GSTs, the majority (nine: Calfi-Delta-V, Calfi-Delta-IX, Calfi-Delta-VIII, Calfi-Delta-I, Calfi-Delta-VI, Calfi-Delta-XI, Calfi-Delta-II, Calfi-Delta-III and Calfi-Delta-X) fell into a single cluster, which was shared with two Delta GSTs from the copepod *T*. *japonicus* (>90% bootstrap support) (Fig 3). The remaining two Delta GSTs (Calfi-Delta-IV and Calfi-Delta-VII) were on separate branches grouped with *D*. *pulex* GSTs with 50% bootstrap support (Fig 3). The second largest diversity of GSTs was found in the Sigma subclass, which grouped into two separate clusters (Fig 3), one of which consisted exclusively of *C*. *finmarchicus* predicted proteins. A single *C*. *finmarchicus* Sigma GST (Calfi-Sigma-VIII [Cf_S8]) did not cluster with any of the others, and was most similar to a *D*. *pulex* Sigma GST, which was also located on its own branch (Fig 3).

### Expression of GSTs during development

**Fig 4:**
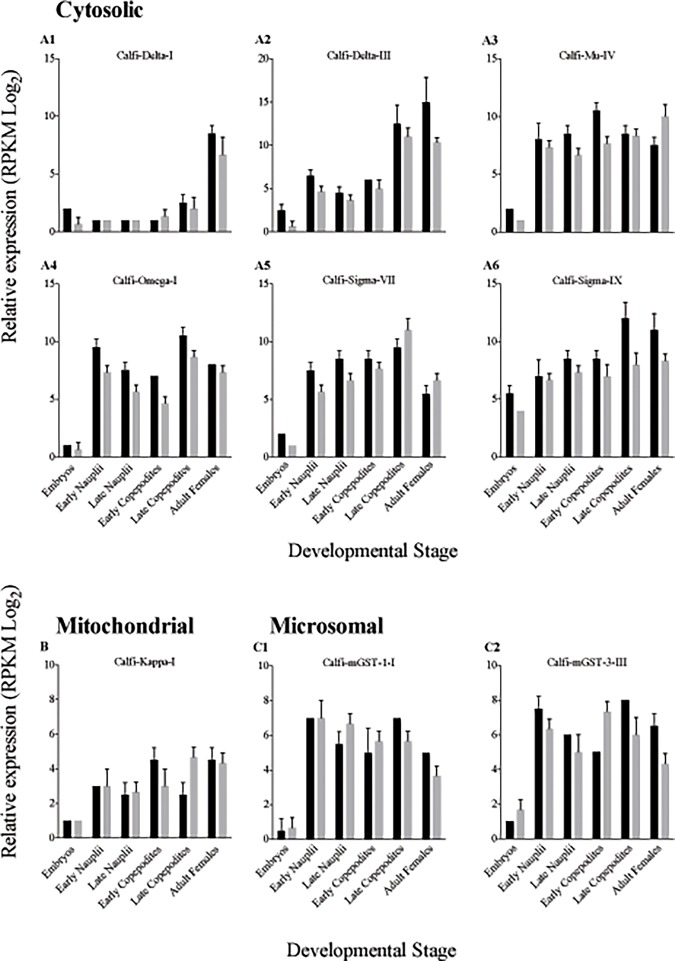
Relative expression of selected *Calanus finmarchicus* cytosolic, mitochondrial and microsomal GST-encoding transcripts across six developmental stages. Relative expression measured in 2011 (black bars) and 2012 (grey bars) for nine GSTs are shown for embryos, early nauplii (NI-II), late nauplii (NV-VI), early copepodites (CI-II), late copepodites (CV), and adult females as RPKM (reads per kilobase per million mapped reads) in Log_2._ (A) Cytosolic GSTs belonging to the Delta (A1-A2), Mu (A3), Omega (A4) and Sigma (A5-A6) subclasses. (B) Mitochondrial Kappa GST class. (C) Microsomal GST subclass 1 (C1) and subclass 3 (C2). Error bars in 2011 (black) are standard deviations of two technical replicates for each stage, while in 2012 (gray) error bars are standard deviations of three biological replicates.

Relative expression of GSTs varied across developmental stages (Fig 4), as well as among GSTs. We observed some differences in relative expression between the two years of sample collection, although in general expression patterns were consistent between years (Fig 4). Expression levels ranged from very low to high with RPKM values ranging between 1 and 14 (Log_2_).

Relative expression in members of the mitochondrial (Calfi-Kappa-I) and microsomal (Calfi-mGST-1-I and Calfi-mGST-3-III) classes was moderately low, but similar across developmental stages except for embryos (Fig 4). Relative expression levels of cytosolic GSTs were more variable across life stages, with most GSTs showing low expression in embryos (Fig 4). Calfi-Delta-III and Calfi-Sigma-IX were the most highly expressed (RPKM Log_2_ between 9 and 11) among the cytosolic GSTs, and peak expression was observed in the adult female and late copepodite stages. In Calfi-Delta-I, expression levels were lower, but showed a similar peak in expression in adult females and late copepodites (Fig 4).

### Gulf of Maine vs. Norwegian Sea: Comparison between two *C*. *finmarchicus de novo* transcriptomes

In addition to the *C*. *finmarchicus* transcriptome generated from material obtained from the Gulf of Maine [34], there is a second *de novo* transcriptome generated from animals from the Norwegian Sea [39]. A total of 39 putative GST-encoding transcripts were retrieved from the Norwegian Sea transcriptome using the GSTs identified in the Gulf of Maine assembly and the known *T*. *japonicus* GSTs as queries (see section above), confirming a similar diversity in GSTs in the two transcriptomes, and hence two populations.

Comparisons between predicted proteins from the two *C*. *finmarchicus* populations found good one-to-one correspondence for the majority of the GSTs in the cytosolic, mitochondrial and microsomal classes (Table 4). Pairwise alignment of the Gulf of Maine query with its Norwegian Sea hit showed that for 36 putative GSTs there was high amino acid conservation (> 90% identity) between the predicted proteins from the two transcriptomes in their regions of overlap (Table 4). This included all predicted GSTs in several cytosolic GST subclasses, *e*.*g*., Sigma (10 proteins), Theta (1 protein), Mu (5 proteins), and Omega (3 proteins), as well as the mitochondrial Kappa GST and the microsomal ones in the mGST-3 subclass (4 proteins; Table 4). The high amino acid identity found between cytosolic GST members of the two populations is in contrast to the amino acid identity between cytosolic GST members within the same subclass, which is lower (see above).

**Table 4:** Comparison between putative *Calanus finmarchicus* glutathione S-transferases (GSTs)* identified via transcriptome mining of two *de novo* assemblies representing populations from the Gulf of Maine [34] and the Norwegian Sea [39].

Class	Subclass	Protein name	Gulf of Maine transcriptome		Norwegian Sea transcriptome		% amino acid identity between proteins
			Transcript accession No.	Deduced protein type	Transcript accession No.	Deduced protein type	
Cytosolic	Delta	Calfi-Delta-I	GAXK01204953	F	GBFB01125513	F	99
		Calfi-Delta-II	GAXK01204968	F	GBFB01087404	F	93
		Calfi-Delta-III	GAXK01204940	F	GBFB01113062	F	98
		Calfi-Delta-V	GAXK01204954	F	GBFB01106634	F	99
		Calfi-Delta-VI	GAXK01204957	F	GBFB01102119	F	93
		Calfi-Delta-VII	GAXK01204947	F	GBFB01126821	F	99
		Calfi-Delta-VIII	GAXK01073468	P	GBFB01085692	F	99
		Calfi-Delta-IX	GAXK01204950	P	GBFB01111155	F	98
		Calfi-Delta-X	GAXK01035521	F	GBFB01031301	P	93
	Theta	Calfi-Theta-I	GAXK01096295	F	GBFB01082889	F	99
	Mu	Calfi-Mu-I	GAXK01204944	F	GBFB01130857	F	100
		Calfi-Mu-II	GAXK01204956	F	GBFB01104663	F	93
		Calfi-Mu-III	GAXK01204948	F	GBFB01069639	F	97
		Calfi-Mu-IV	GAXK01204952	F	GBFB01171394	F	98
		Calfi-Mu-V	GAXK01204958	F	GBFB01086262	P	200
	Omega	Calfi-Omega-I	GAXK01204960	F	GBFB01112247	F	94
		Calfi-Omega-II	GAXK01164502	P	GBFB01122297	P	98
		Calfi-Omega-III	GAXK01016325	P	GBFB01061154	P	99
	Sigma	Calfi-Sigma-I	GAXK01204964	F	GBFB01117919	F	92
		Calfi-Sigma-II	GAXK01204943	F	GBFB01053851	F	99
		Calfi-Sigma-III	GAXK01204943	F	GBFB01057086	F	99
		Calfi-Sigma-IV	GAXK01204959	F	GBFB01080562	F	97
		Calfi-Sigma-V	GAXK01204951	F	GBFB01211675	P	99
		Calfi-Sigma-VI	GAXK01204961	P†	GBFB01107763	F	96
		Calfi-Sigma-VII	GAXK01204946	F	GBFB01147064	F	100
		Calfi-Sigma-VIII	GAXK01204966	F	GBFB01103677	P	98
		Calfi-Sigma-IX	GAXK01204949	F	GBFB01105091	F	99
		Calfi-Sigma-X	GAXK01204945	F	GBFB01125239	F	97
	Zeta	Calfi-Zeta-I	GAXK01204941	F	GBFB01157033	P	100
		Calfi-Zeta-II	GAXK01084871	P	GBFB01237836	P	100
Mitochondrial	Kappa	Calfi-Kappa-I	GAXK01046934	F	GBFB01121887	F	99
Microsomal	mGST-1	Calfi-mGST-1-I	GAXK01178264	F	GBFB01067142	F	100
	mGST-3	Calfi-mGST-3-I	GAXK01204963	F	GBFB01089387	F	99
		Calfi-mGST-3-II	GAXK01204967	F	GBFB01094405	F	100
		Calfi-mGST-3-III	GAXK01204955	F	GBFB01076955	F	100
		Calfi-mGST-3-IV	GAXK01204962	P†	GBFB01082093	F	99

*GST transcripts listed showed >90% amino acid identity between proteins.

†In Table 2 these proteins were identified as putative full-length because flanked by a “methionine’ at the N-terminal, and a “stop” codon at the C-terminal, and by the conservation of structural domains. However, alignment of each protein with its counterpart from the “Norwegian Sea” transcriptome suggests that they are partial proteins.

The second transcriptome not only confirmed the presence of the GSTs, but also provided additional data. In four cases (Calfi-Delta-VIII, Calfi-Delta-IX, Calfi-Sigma-VI and Calfi-mGST-3-IV) the transcripts from the Norwegian Sea transcriptome predicted full-length proteins, while the transcripts identified from the Gulf of Maine assembly encoded only partial ones (Table 4). In another three cases, genetic differences between the two transcriptomes were larger than expected. In the first case, there appeared to be an additional Omega transcript in the Norwegian Sea transcriptome (Table 5). Protein translation and structural analyses confirmed that the protein was full-length and possessed the typical structural hallmarks (N- and C-terminal domains) of a cytosolic Omega subclass member. A reciprocal BLAST search of the non-redundant arthropod protein database identified its top protein hit as an Omega GST from the insect *L*. *migratoria* (**Accession No.** **AFK10494**). Pairwise comparison of this fourth Omega GST with the other three Omega GSTs (Table 4) showed only 30%–37% amino acid identity, suggesting that this transcript represents an additional gene in this subclass. A search of the Gulf of Maine transcriptome using this fourth Omega protein as the query yielded a short nucleotide sequence (504 base pairs) which encoded a partial protein that was 99% identical in sequence to the corresponding portion of the query, confirming the presence of this Omega GST in both transcriptomes (Table 5). Thus, *C*. *finmarchicus* appears to have four genes encoding GSTs in the Omega subclass.

**Table 5:** Putative *Calanus finmarchicus* glutathione S-transferases (GSTs) showing differences between the Gulf of Maine [34] and the Norwegian Sea [39] *de novo* assemblies.

Type of variation	Protein name	Gulf of Maine transcriptome		Norwegian Sea transcriptome		% amino acid identity between proteins
		Transcript accession No.	Deduced protein type	Transcript accession No.	Deduced protein type	
Genetic variation	Calfi-Delta-IV	GAXK01204965	F	GBFB01111154	F	48
	Calfi-Delta-XI	GAXK01204939	F	GBFB01141707	F	88
Additional gene	Calfi-Omega-IV	GAXK01138968*	P	GBFB01119512	F	99

*This transcript was not identified as encoding a GST protein in the original screening of the Gulf of Maine transcriptome, but rather was detected via a query with its Norwegian Sea counterpart.

Large differences in amino acid sequences were found for two Delta GSTs, Calfi-Delta-IV and Calfi-Delta-XI, when paired with their top hits in the Norwegian Sea transcriptome (< 90% identity) (Table 5). In one case (Calfi-Delta-IV), amino acid identity was only 48% between the Gulf of Maine protein and its Norwegian Sea counterpart (Table 5). This level of amino acid identity is similar to the one we observed among different members of the same subclass (usually 30%–50%), suggesting that this cytosolic GST may be derived from a separate gene, and thus represent a 12^th^ gene in the Delta sub-class. More difficult to interpret is the 88% amino acid identity found between Calfi-Delta-XI and its Norwegian counterpart (Table 5); the two proteins did not fall into what had been previously defined as “good one-to-one correspondence” (≥ 90% amino acid identity in region of overlap) but, nevertheless, they shared more then the expected amino acid identity (30%–50%) between subclass members. Thus, if these two Delta GSTs are derived from the same gene, they show significant genetic divergence between the two populations.

In summary, comparison of the two transcriptomes yielded a more complete set of predicted GSTs for *C*. *finmarchicus*. By combining the two data sets, we have predictions for 36 full-length proteins (88%) and five partial ones. Thirty-nine of these GSTs showed good to excellent amino acid identity (88%-100%) between transcriptomes, and hence populations. Two proteins were found in the Gulf of Maine transcriptome but not in the Norwegian Sea transcriptome. Two additional cytosolic genes were predicted from the Norwegian Sea transcriptome that were absent in the Gulf of Maine dataset, bringing the gene diversity in the Delta subclass to 12 and the Omega subclass to 4 predicted proteins. Based on these two transcriptomes, *C*. *finmarchicus* is predicted to have a total of 41 GSTs.

## Discussion

The GSTs belong to a gene superfamily that is present in both prokaryotes and eukaryotes [7]. In the arthropods, this superfamily is characterized by multiple gene duplications, leading to a diverse set of genes, some of which have been shown to be rapidly evolving in response to natural selection, such as exposure to new insecticides [57]. Genome sequencing and bioinformatics-based data mining have been a powerful strategy for the discovery and characterization of GSTs. In insects, the number of GST genes varies widely with 13 genes reported in *Apis mellifera*, 23 in *B*. *mori*, 31 in *A*. *gambiae*, 40 in *D*. *melanogaster* and 41 in *T*. *castaneum* [53]. Among the crustaceans, the cladoceran *D*. *pulex* is the only species with a sequenced genome, and its GST superfamily consists of 31 genes [14]. Here, we identified putative GSTs belonging to the cytosolic (34 proteins), microsomal (6 proteins) and mitochondrial (1 protein) classes in the calanoid copepod *C*. *finmarchicus* by mining two *de novo* transcriptomes using a workflow that included reciprocal BLAST and protein structural analyses. This number is much higher than the twelve GSTs that were identified and classified by *in silico* EST mining in *T*. *japonicus* ([15], Roncalli, unpublished), and the 12 GSTs identified in a search of publicly available ESTs of *C*. *clemensi* (search completed 12/04/2014; Roncalli, unpublished). However, these may be underestimates given the limited size of the EST databases available for *T*. *japonicus* and *C*. *clemensi*. More recently, transcriptome shotgun assemblies have been made available for several copepods, including *L*. *salmonis* and *C*. *rogercresseyi*. Searches for “glutathione S-transferase” in these TSA databases on NCBI (search date: 12/04/2014) resulted in 34 transcripts annotated as encoding GST proteins in *L*. *salmonis* (**Bioproject No.** **PRJNA73429**) and 35 in *C*. *rogercresseyi* (**Bioproject No.** **PRJNA234316**). Yang et al. [58] reported 31 GST proteins in the *de novo* transcriptome of the calanoid *Calanus sinicus*, but to date, these data are not publicly accessible. None of these studies included annotations by GST class or subclass, or protein structural analyses. However, in general, it appears that the number of GST genes in these copepod species exceeds 30 based on automated annotations of TSA data (e.g. [58]).

Although two conserved domains characterize all cytosolic GSTs irrespective of subclass [4], these proteins are nevertheless highly diverse. In the insects, cytosolic GST members belonging to the same subclass within a species have typically 40%-50% amino acid identity [59]. We found a similar pattern in *C*. *finmarchicus*, where even cytosolic GSTs with identical top hits were quite different from each other, with amino acid identity ranging from 27%-60%, supporting the conclusion that each of the 34 cytosolic GSTs represents a transcript from a separate gene. In contrast, when we compared GSTs obtained from two separate transcriptomes, the predicted proteins were much more similar. Twenty-two (56%) of the predicted proteins were 99%-100% identical, while seventeen showed moderate differences with 88%-98% identity in amino acid sequence in the region of overlap. The transcriptomes were generated from mRNA from individuals from two populations of *C*. *finmarchicus* (Gulf of Maine and Norwegian Sea) that are separated by over 4,000 km, and these two populations are mostly isolated from each other [60,61]. Population genetic studies suggest two to four genetically distinct *C*. *finmarchicus* populations across the North Atlantic with no direct genetic exchange between the Gulf of Maine and Norwegian Sea [60,61]. However, there is evidence for genetic connectivity via the central North Atlantic with genetic exchanges between this *C*. *finmarchicus* population and the ones in the Labrador Sea/Gulf of Maine in the western Atlantic and the Norwegian Sea in the eastern Atlantic [61]. Thus, the observed differences in GST protein predictions from the two transcriptomes are not surprising, given that genes in this superfamily are often under natural selection and have been shown to evolve rapidly in other arthropods [4,6]. However, whether the genetic variation in *C*. *finmarchicus* represents differences in function in response to habitat-specific selection has yet to be determined.

Glutathione S-transferases are best known for their role in detoxification of xenobiotics, although other functions have been described [62]. Given the diversity of environmental toxins and pollutants, and their variable levels of toxicity, it has been hypothesized that the need to metabolize a variety of xenobiotics has driven the expansion of the cytosolic GSTs [62]. In insects, the subclasses Delta and Epsilon are responsible for the removal of chemical compounds produced by either their food or by pesticides [63,64]. The number of GSTs in the Delta subclass is variable: some species have just a few, *e*.*g*., *A*. *mellifera* (2), *B*. *mori* (5) and *T*. *castaneum* (3), while others have over ten, *e*.*g*., *Acyrthosiphon pisum* (16), *A*. *gambiae* (17) and *D*. *melanogaster* (11). In *C*. *finmarchicus*, the Delta GST subclass is large with a total of 12 different proteins predicted. If the function of the Delta GSTs in *C*. *finmarchicus* is similar to that of the insects, extensive gene duplication may have occurred in response to environmental toxins encountered by this copepod. *C*. *finmarchicus* is a filter feeder and it consumes a variety of microplankton including diatoms, dinoflagellates, flagellates, ciliates and protozoans [65,66]. Many common food types such as dinoflagellates and diatoms are known to produce toxic secondary metabolites as defense against predators, competitors and pathogens [67]. Although it has been demonstrated that copepods can feed selectively [68] and thus might be able to avoid consuming toxic species, there is good evidence that copepods, including *C*. *finmarchicus*, ingest toxic species during natural blooms [69].

In the Gulf of Maine, *C*. *finmarchicus* frequently encounters algal blooms dominated by the toxic dinoflagellate *Alexandrium fundyense*, known for the production of saxitoxins, which are highly toxic to humans, birds, fishes and marine mammals [20,70,71]. *C*. *finmarchicus* ingests *A*. *fundyense* with no detrimental effects on its survival [72–75]. Spring blooms dominated by diatoms in the genera *Thalassiosira*, *Skeletonema* and *Chaetoceros* spp. are common in both the Gulf of Maine and the Norwegian Sea [76,77]. These diatom genera are known for their production of oxylipins, which are toxic at high concentrations to other copepods, such as the congener *C*. *helgolandicus* [78–80]. Thus, *C*. *finmarchicus* inhabiting either the Gulf of Maine or the Norwegian Sea are likely to experience a wide range of natural toxins during their life cycle given a diet that includes phytoplankton species producing a variety of metabolites. The high gene diversity in the Delta GST subclass, which is involved in detoxification, may represent a fitness advantage for *C*. *finmarchicus*.

Sigma represents the second largest subclass with 10 putative GSTs in *C*. *finmarchicus*. A similar number of Sigma GSTs are present in the cladoceran *D*. *pulex* [14], but the diversity in insects is typically lower and ranges from a single gene (*D*. *melanogaster* and *A*. *gambiae*) to six (*A*. *pisum*) or seven (*T*. *castaneum*) [53]. The Sigma GST subclass plays an important role in the protection against oxidative stress in insects [5]. However, it is less clear why this subclass is so diverse in the crustaceans, and the function of individual Sigma GSTs has yet to be investigated even in model species like *D*. *pulex*. The phylogenetic relationship among the Sigma GSTs (Fig 3) showed species-specific clustering of the *D*. *pulex* Sigma GSTs and the majority (6) of the *C*. *finmarchicus* Sigma GSTs. Further studies are needed to determine whether high diversity in Sigma GSTs is common in all crustaceans, and to establish their physiological functions.

In addition to their role in detoxification of exogenous compounds, GSTs play a role during development [81]. A peak in expression was found in the pre-pupal and pupal stages in Sigma GSTs in the insects *Mayetiola destructor*, *Lucilia cuprina* and *Agrilus planipennis*, presumably in response to an increase in metabolic activity and apoptosis associated with the morphological changes that occur during these periods [82–84]. Similarly, detoxification from byproducts produced during metamorphosis may explain high relative expression of Delta GSTs in the insects *D*. *melanogaster*, *A*. *planipennis* and *Nilaparvata lugens* during the pupal stage [56,84]. Copepods, like insects, undergo a significant morphological rearrangement between the 6^th^ naupliar and 1^st^ copepodite stages [85]. This change in morphology occurs during a molt cycle, and does not involve a pupal stage as in the insects. No significant changes in expression level in either Delta or Sigma GST-encoding transcripts correlated with this transition. Instead, we found highest expression of cytosolic GSTs in the CV and adult female stages. One possible explanation is that in our sample, these late stages were field collected, and thus they had been exposed to a mix of phytoplankton species, while the early developmental stages were laboratory reared on a single algal species [34]. The difference in expression may be related to exposure to natural toxins in the field-collected animals.

## Summary and Conclusion

Using two *de novo* assembled transcriptomes, transcripts encoding 41 distinct GST proteins were identified for the copepod *C*. *finmarchicus*. The deduced proteins included members of the cytosolic, mitochondrial and microsomal classes, with the highest diversity observed in the cytosolic class. The transcripts/proteins likely represent the products of distinct genes, and if true, the diversity of GST in *C*. *finmarchicus* exceeds or rivals that described for insects and other crustaceans. The food sources and life history of *C*. *finmarchicus* are likely factors driving selection for this diversity, as this copepod is commonly exposed to a wide variety of natural toxins, and hence multiplicity in detoxification pathway proteins may well be key to their survival. Characterization of the GST superfamily in *C*. *finmarchicus* opens opportunities for functional studies of detoxification, and provides a diverse set of biomarkers for this species. These biomarkers will likely be useful for future studies evaluating ecosystem health and organism-environment interactions in the North Atlantic, an area that is regularly challenged by a variety of natural and anthropogenic stressors.
